# Visual analogue scale (VAS) as a monitoring tool for daily changes in asthma symptoms in adolescents: a prospective study

**DOI:** 10.1186/s13223-017-0196-7

**Published:** 2017-04-28

**Authors:** Hyekyun Rhee, Michael Belyea, Jennifer Mammen

**Affiliations:** 1 University of Rochester, School of Nursing, 601 Elmwood Avenue, Box SON, Rochester, NY 14642 USA; 2 Arizona State University, College of Nursing and Health Innovation, 500 N. 3rd Street, Phoenix, AZ 85004 USA

**Keywords:** Visual analogue scale, Asthma symptom monitoring, Adolescents, Symptom diary, Asthma control

## Abstract

**Background:**

Success in asthma management hinges on patients’ competency to detect and respond to ever-changing symptom severity. Thus, it is crucial to have reliable, simple, and sustainable methods of symptom monitoring that can be readily incorporated into daily life. Although visual analogue scale (VAS) has been considered as a simple symptom assessment method, its utility as a daily symptom monitoring tool in adolescents is unknown. This study was to determine the concurrent validity of VAS in capturing diurnal changes in symptoms and to examine the relationships between VAS and asthma control and pulmonary function.

**Methods:**

Forty-two adolescents (12–17 years old) with asthma completed daily assessment of symptoms twice per day, morning and bedtime, for a week using VAS and 6-item symptom diary concurrently. Asthma control was measured at enrollment and 6 month later, and spirometry was conducted at enrollment. Pearson correlations, multilevel modeling and regression were conducted to assess the relationships between VAS and symptom diary, asthma control and FEV1.

**Results:**

Morning and evening VAS was positively associated with symptom diary items of each corresponding time frame of the day (r = 0.41–0.58, p < 0.0001). Morning VAS was significantly predicted by morning diary data reflecting nocturnal wakening (β = 2.13, p = 0.033) and morning symptoms (β = 4.09, p = 0.002), accounting for 57% of the total variance of morning VAS. Similarly, changes in four evening diary items, particularly shortness of breath (β = 2.60, p = 0.028), significantly predicted changes in evening VAS, accounting for 55% of the total variance. Average VAS scores correlated with asthma control (r = 0.65, p < 0.001) and FEV1 (r = −0.38, p = 0.029), and were predictive of asthma control 6 months later (β = 0.085, p = 0.006).

**Conclusions:**

VAS is a valid tool capturing diurnal changes in symptoms reflected in a multi-item symptom diary. Moreover, VAS is a valid measure predicting concurrent and future asthma control. The findings suggest VAS can be a simple alternative to daily dairies for daily symptom monitoring, which can provide invaluable information about current and future asthma control without substantially increasing self-monitoring burdens for adolescent patients.

*Clinical Trial Registration* NCT01696357. Registered 18 September 2012

## Background

Asthma symptom monitoring incorporated in daily routines is essential to successful asthma self-management [1–3]. Symptom monitoring is linked to fewer cases of asthma exacerbation and acute care visits, as well as better functional outcomes and higher quality of life in children and adolescents [4]. Recognition of symptoms through monitoring allows patients to take necessary management actions (e.g., adjusting medication, altering activity level, avoiding or minimizing triggers, or seeking assistance). Moreover, patients’ symptom monitoring is an essential source of information in implementing guideline based treatment.

Expert Panel Report 3 (EPR-3) by the National Heart, Lung and Blood Institute [3] recommends that each patient be taught to recognize symptom patterns indicating inadequate asthma control. The need for a patient completed monitoring tool that can capture the variability and changing nature of asthma between office visits has been recognized [5, 6]. To address the need, symptom diaries have been suggested especially for those with uncontrolled or persistent asthma, as it can aid in the identification of asthma of higher severity [3, 7]. Asthma diaries completed on a daily basis minimize recollection errors [8], and are a better means for identifying patients with persistent asthma compared to retrospective reports of usually previous 4 weeks, as in a periodic self-assessment form completed at the time of an office visit (e.g., asthma control test) [7].

Structured daily diaries often consist of multiple items assessing various symptoms and asthma related impairments, which can be burdensome to children and adolescents, resulting in poor adherence. This calls for an alternative symptom monitoring strategy that is simple and conducive to daily use by young patients. A visual analogue scale (VAS) can be the alternative to multiple item diaries for daily symptom monitoring. VAS has been found useful in assessing patient’s subjective experience or perception of a variety of clinical phenomena [9]. VAS scores have been shown to be associated with varying levels of asthma control [10–12] and pulmonary function [11, 12]. A simple VAS performed better in capturing asthma severity compared to other structured assessment methods of multiple categories measuring the same construct [13]. For being a single item with minimal wording, VAS requires little time or literacy to complete. These advantages render VAS suitable for daily use for symptom monitoring, particularly in pediatric patients.

The extent to which the VAS performs as an alternative to multiple item diaries for a daily symptom assessment tool for adolescents remains to be explored. In particular, demonstrating its capacity to capture diurnal and day-to-day variations in symptoms would be critical to be considered as a symptom monitoring tool. The purpose of this study was to determine the concurrent validity of VAS in capturing diurnal changes in symptoms and to examine the relationships between VAS and asthma control and pulmonary function.

## Methods

### Study sample and setting

This study was the secondary analysis of data originally collected for a study that examined the validity of a newly developed automated device monitoring asthma symptoms [14]. Eligibility criteria included age 13 through 17, having physician-diagnosed asthma at least 1 year, and ability to understand spoken and written English. Those with other diagnoses producing asthma-like symptoms (e.g., cardiac disease and cystic fibrosis) were excluded. Subjects were recruited from the pediatric emergency department (ED) and outpatient clinics (primary practice and pediatric pulmonary specialty clinic) in a major university medical center located in the Northeastern U.S. Of a total of 42 participants, the majority (69%) were recruited from the emergency department, and the remaining were from the study flyers (24%) and clinician referrals (8%).

### Study measures

#### Visual analogue scale (VAS)

The VAS was 100 mm long with 3 anchors dividing 3 zones (green, yellow, and red). For each symptom, the green zone (0–20 mm) labeled “no symptoms”, the yellow zone (21–50 mm) “mild symptoms” and the red zone (50–100 mm) “very bad symptoms” [13, 15]. Teens marked any point on the zone according to their perception of symptoms. The distance between the 0 mm mark and the placement of the “X” was measured to provide a numeric interpretation of their symptom perception. VAS was used twice, morning (VAS-am) and bedtime (VAS-pm).

#### Asthma Control Diary (ACD)

This 6-item diary measures the levels of symptoms on a 7-point scale from zero (no symptom) to 6 for consistent symptoms [16]. The diary data were collected electronically using an iPod to increase participants’ adherence and convenience. By sending automatic reminders and restricting the time of entry, the electronic diaries were useful in ascertaining adherence and reinforcing proper entry time, thereby reducing the recollection bias and risk of fabrication. Morning questions (2 items) pertained to nocturnal wakening and morning symptoms, and bedtime questions (4-items) assessed the degree of activity limitation, shortness of breath, and wheeze they experienced during the day and use of short-acting beta agonist (SABA) in previous 24 h. Each item was evaluated separately in the analysis.

#### Forced expiratory volume in one second (FEV1)

Spirometry test (KoKo^®^: Pulmonary Data Service; Louisville, CO, USA) was conducted on the first day of the 7-day trial to obtain FEV1 in accordance with the American Thoracic Society standards [17].

*Asthma control questions* consisting of 4 questions were devised for the study based on four impairment based asthma control criteria provided by EPR3. The criteria including the frequency of the limitation of daily activity, nocturnal wakening, asthma symptoms and use of SABA in the past 4 weeks were measured on a 5-point scale. Asthma control questions were administered at enrollment and at the 6-months follow-up. A total score was computed; higher scores indicating poor asthma control.

*Demographic and asthma*-*related information* was collected, including gender, age, race, annual family income, years with asthma diagnosis and current medications.

### Data collection procedure

Institutional Review Board within the academic institution affiliated with the principal investigator (HR) reviewed and approved the study for human subject protection. Informed consent was obtained from parents and assent from adolescents prior to data collection. At enrollment, a demographic form, asthma control questions and spirometry were administered. Adolescents completed a paper form VAS twice a day, morning and bedtime, for 7 days. Simultaneously, electronic asthma diaries were completed using an iPod programed to push morning and evening questions at the designated timeframes each day for 7 days. Automatic reminders were sent until the completion of scheduled diary questions within the timeframe. Asthma control questions were repeated at the 6-month follow up.

### Data analysis

Pearson correlations were computed to assess the relationships between VAS and ACD, asthma control and FEV1. A multilevel regression model for longitudinal data was used to assess the relationships between VAS and daily asthma symptoms reported in ACD measured twice a day for seven consecutive days. Morning and evening VAS measures were analyzed using two hierarchical models: (1) a model with time varying covariates, and (2) a final model with the addition of the demographic covariates. Diary symptoms were grand mean centered, where 0 represented the average level of the measure, and entered simultaneously into the model.

In the first model, asthma symptoms as time-varying predictors of VAS were entered into the model. In these models, VAS was modeled with an intercept, linear slope, and with the time-varying covariates entered simultaneously (nocturnal wakening and morning symptoms for morning VAS and activity limitation, shortness of breath, wheezing, and SABA use for evening VAS). Using daily asthma symptoms as time-varying covariates tests whether they can explain changes in the overall trajectory of VAS. Such tests would examine whether VAS and asthma symptoms measured at the within-person level changed together.

In the second model, age, sex, and race were entered as time fixed demographic variables. The introduction of time fixed covariates tests whether the association between VAS and asthma symptoms persists after controlling for the demographic variables. Multilevel regression analysis, using all available data from each participant, was conducted using the Mixed procedure in SAS. Ordinary least squares regression analysis was conducted to examine the extent to which VAS predicted asthma control after 6 months. All statistical analyses were conducted in SAS (v.9.3). The level of significance was set at p < 0.05 for all tests.

## Results

Forty-two adolescents (mean age 15.2 years; SD = 1.5) participated: 60% (n = 25) females, 57% (n = 24) minority and 52% (n = 22) with annual household income less than $30,000. Based on the EPR-3 classification criteria, 36% (n = 15) and 33% (n = 14) of the sample reported not well controlled and very poorly controlled asthma, respectively.

### Correlations between VAS and Asthma Control Diary Questions

The relationships between average VAS for morning and evening and each time-corresponding diary data obtained for 7 days were examined in 41 persons. One participant’s electronic diary data were lost due to unknown technical glitches. Table 1 displays the summary of correlation coefficients between VAS and each of items of ACD. Significant correlations were found between VAS and ACD, ranging from r = 0.41 to 0.58.

**Table 1:** Correlations between VAS and asthma diary items

	VAS-am	VAS-pm
Morning diary items		
Nocturnal wakening	0.45***	
Morning symptoms	0.55***	
Evening diary items		
Activity limitation		0.41***
Shortness of breath		0.52***
Wheezing		0.58***
Short acting beta agonist use		0.42***

*** p = <.0001

### Morning VAS and morning diary variables

Table 2 displays the descriptive statistics of morning VAS (VAS-am) data over 7 days.

**Table 2:** Descriptive statistics for morning VAS

Time point	N	Mean	SD	Skewness	Kurtosis	Range
VAS Day 1	34	19.18	19.85	1.69	2.95	0–82
VAS Day 2	41	18.41	16.77	1.20	0.91	0–63
VAS Day 3	41	18.95	19.82	1.79	3.25	0–84
VAS Day 4	41	16.98	15.50	1.09	0.84	0–65
VAS Day 5	41	15.93	17.24	1.87	3.86	0–75
VAS Day 6	41	17.05	17.50	1.17	0.48	0–64
VAS Day 7	41	15.59	15.21	0.70	−0.96	0–50
Average	41	17.33	14.58	0.79	−0.49	0–50.71

Multilevel regression models for the 7 measurement points were conducted. Model 1 (Table 3) was computed to examine the relationship between VAS-am and two morning diary items, nocturnal wakening and bad morning symptoms, as time varying covariates.

**Table 3:** Morning VAS predicted by the morning diary items as time varying covariates

	Model 1			Model 2		
	Standard			Standard		
	Estimate	SE	p	Estimate	SE	p
Effect						
Intercept	18.13	2.17	≤.001	−0.70	−0.70	0.974
Time	−0.24	0.33	0.479	−0.32	0.34	0.360
Nocturnal wakening	2.21	0.98	0.024	2.13	0.99	0.033
Morning symptoms	3.79	1.20	0.002	4.09	1.29	0.002
Age				0.81	1.34	0.557
Sex (1 = female)				5.18	4.25	0.224
Race (1 = non-white)				5.77	4.26	0.177

The average starting or initial level of morning VAS was 18.13 (t = 8.35, p = 0.001). The linear slope (time) was not significant indicating that the level of reported VAS-am was relatively stable for each individual for 7 days as a whole. However, tests of the random effects indicated significant individual differences in the intercepts or starting values for VAS-am (z = 3.73, p < 0.0001) and individual differences in its slopes or linear change over time approached significance (z = 1.46, p < 0.07). Changes in nocturnal wakening (β = 2.21, p = 0.024) and morning symptoms (β = 3.79, p = 0.002) were significantly related to changes in morning VAS. Including age, gender and race as covariates (Model 2, Table 3) did not substantially change these relationships. Approximately 57% of the total variance of VAS-am scores was accounted for by Model 2.

### Evening VAS and evening diary variables

Table 4 displays the means and distributions for evening VAS (VAS-pm) over 7 days.

**Table 4:** Descriptive statistics for evening VAS

Time point	N	Mean	Std D	Skewness	Kurtosis	Range
VAS Day 1	41	18.80	19.82	1.77	3.30	0–87
VAS Day 2	40	19.83	21.13	1.84	4.12	0–100
VAS Day 3	41	20.95	20.36	1.24	0.95	0–77
VAS Day 4	40	17.98	17.20	1.49	2.45	0–75
VAS Day 5	40	15.10	15.13	1.31	1.33	0–63
VAS Day 6	41	18.05	17.84	1.18	0.89	0–74
VAS Day 7	41	18.37	17.37	1.08	0.65	0–70
Average	41	18.44	15.83	1.00	0.27	0–62.86

Model 1 examined the relationship between VAS-pm and the evening diary variables of limited activity, shortness of breath, wheeze, and SABA use as time varying covariates. Model 1 (Table 5), indicates that on average, the starting or initial level of VAS-pm for participants was 18.58. The linear trend for the group as a whole was not significant indicating that the overall average VAS-pm was relatively stable.

**Table 5:** Evening VAS predicted by the evening diary items as time varying covariates

	Model 1			Model 2		
	Standard			Standard		
	Estimate	SE	p	Estimate	SE	p
Effect						
Intercept	18.58	2.83	<0.001	−19.62	19.10	0.311
Time	−0.13	0.49	0.795	−0.08	0.52	0.874
Activity limitation	0.11	0.89	0.904	0.21	0.93	0.821
Shortness of breath	2.27	1.11	0.042	2.60	1.18	0.028
Wheezing	2.27	1.20	0.060	2.11	1.24	0.089
SABA use	−0.45	1.67	0.786	−0.95	1.67	0.571
Age				1.97	1.21	0.104
Sex (1 = female)				9.00	3.81	0.019
Race (1 = non-white)				3.41	3.83	0.375

Shortness of breath had a significant effect on VAS-pm (β = 2.27, p = 0.04) after controlling for other items, such that changes in shortness of breath predicted changes in VAS-pm scores above and beyond other evening diary items. Wheezing approached significance. Including age, gender, and race as covariates (Model 2) further reduced the effect of wheezing, but did not change the relationship between shortness of breath and VAS-pm. Females reported higher VAS-pm. The total variance accounted for in VAS-pm scores by Model 2 was approximately 55%.

### VAS and asthma control and FEV1

Adjusting for age, gender and race, average VAS scores from 7 days correlated with asthma control questions (r = 0.65, p < 0.001) and FEV1 (r = −0.38, p = 0.029). Average VAS was predictive of asthma control at 6 months (β = 0.085, p = 0.006) after controlling for age, gender and race. VAS along with demographic variables accounted for 33% of variance of asthma control at 6-months.

## Discussion

This study demonstrates that VAS can be a reasonable alternative to symptom diaries for daily symptom monitoring in adolescents with asthma. To our knowledge, this is the first study to provide evidence for VAS’ capacity to reflect diurnal and daily variation in asthma symptoms. In an earlier cross-sectional study, VAS appropriately differentiated between good, usual and bad breathing days in children with asthma [18]. A prospective study also supported VAS as an instrument that could reasonably detect symptom variations between two discrete observation points with 2 weeks apart in adult patients with allergic rhinitis [19]. Our findings provide more specific evidence that VAS can capture symptom fluctuations occurring not only day-to-day but also within each day.

Consistent with earlier studies [11, 20, 21], we found moderate correlation between VAS and pulmonary function, supporting VAS as a tool for measuring airway obstruction. Validity and clinical usefulness of VAS has often been evaluated in comparison to an objective assessment of airway obstruction such as FEV1. Studies have shown correlations between the degrees of symptoms indicated on VAS and FEV1 in adults [12, 22] and children with asthma [11, 23]. The relationships between VAS and pulmonary function do not appear to be affected by age and gender of children [11]. In fact, symptom perception measured on VAS reliably correlated with pulmonary function in children with asthma across a broad age ranging from 5 to 15 years [24]. Furthermore, in an earlier study [21], VAS was able to discriminate children with bronchial obstruction (FEV1 <80% predicted) from those with normal lung function through the demonstration of significant group differences in VAS scores at a single point. Also, changes in symptom perceptions after use of bronchodilator in children with asthma were also adequately captured by VAS, which was suggestive of bronchial reversibility [21]. Based on the evidence, the authors advocated for the potential application of VAS in establishing an asthma diagnosis in the absence of spirometer in some office settings [21]. Overall, accumulated evidence including ours is overwhelmingly in favor of VAS of symptom perception as a proxy measure of airway obstruction.

As in a previous study [25], we found a moderate to strong correlation between VAS and symptom control. It is important to recognize that traditional measures of symptom control often fail to capture the full extent of symptoms, particularly in adolescent populations, resulting in underestimation of symptoms [26, 27]. Therefore, the lack of decisive correlation between VAS and a conventional measure of asthma control should not be interpreted as an indication of inadequacy of the VAS. On the contrary, it is equally likely that unexplained variance between the VAS and the conventional asthma control measure may have been due to the conventional measure’ limited capacity to fully represent the construct of symptom control. Nonetheless, demonstrated correlations between VAS and symptom diary items were close to or well above 0.5, indicating large effect size [28]. Similar to our findings, a large cross-sectional epidemiological study of nearly 30,000 adult patients demonstrated that VAS measured symptom severity accurately and predicted asthma control as defined by Global Initiative for Asthma [10]. This earlier study also showed no differences in VAS scores between patient-rated versus physician-rated, supporting the reproducibility of the measure across different raters. Furthermore, we found that VAS was predictive of asthma control 6 months later. To our knowledge, this is the first study providing the evidence of predictive validity of VAS longitudinally. The possibility of gauging future asthma morbidity using simple VAS has important clinical implications as it could help patients and clinicians determine plans for long-term treatment.

Taken all together, VAS scores could be a simple, reliable indicator of asthma control in adolescents, when used daily. VAS is easily understood and can be a useful tool in improving children’s perceptual ability [29]. However, there are mixed reports regarding when VAS would be useful and valid in asthma monitoring. Some reported that VAS more adequately measured symptom control when severity is low [20, 30]. Given that accuracy of symptom perception in children and adolescents shows a marked decrease when symptom severity elevates [27, 31], perhaps VAS may be more appropriate for those with intermittent to mild severity. Conversely, others provided evidence demonstrating VAS to be more useful in those with moderate to severe asthma [12] or airway obstruction (FEV1 <80%) [11]. Similarly, in another study [32] VAS scores reflected peak exploratory flow rates more adequately when children experienced actual symptoms compared to symptom-free times, hence supporting use of VAS only for moderate to severe cases of asthma. In order for VAS to be considered as a daily symptom monitoring tool, however, it is essential that it be sufficiently sensitive to wide range of symptom severity from low to high. Although this study offers a glimpse of the possibility that VAS could capture the broader spectrum of symptom severity, further research is required to provide more conclusive evidence in a large sample of adolescents with differing levels of symptom control.

VAS is a simple and straightforward method to assess daily variations of asthma symptoms and the degrees of asthma control and airway obstruction. The simplicity makes the measure less dependent on users’ literacy or attention capability and requires very little time to complete. Nonetheless, by allowing responses to vary along the line of continuum, VAS could adequately assess and quantify subtle changes in an individual’s perception about a subjective attribute such as symptoms over time [9, 33]. Evidence that VAS effectively captured positive changes in asthma condition responding to bronchodilation [21] provides further support for the tool’s usefulness in assessing treatment effects and preventing overestimation of asthma control through improved symptom perception [34]. These benefits render VAS a practical daily symptom assessment tool that can potentially maximize users’ long-term compliance, through which symptom perception can be improved and asthma morbidity can be prevented or detected at an earlier stage.

This study is subject to several limitations germane to the study design. First, a small convenient sample of adolescents with asthma limits the generalizability of the findings. It is also worth mentioning that despite a large portion of our sample having been recruited from the emergency department, average VAS scores were generally low indicating mild symptoms albeit with wide variations. In USA, low income families tend to use the ED for usual asthma care or management that could have been handled at primary care practices [35, 36]. Hence, ED visits do not always suggest higher symptom severity in such populations. A large-scale study using a sample representing a broad spectrum of symptom severity is warranted to assure the replicability of our findings prior to broad clinical implementation of VAS as a symptom monitoring tool. Second, our relatively brief observation period (7 days) prevented us from establishing the validity of VAS for an extended period during which symptom variations may become more pronounced. Third, we were unable to assess changes in VAS scores responding to treatment (e.g., short-term vs. long-term medication) during the observation period. Strategically timed administration of VAS before and after specific medication is needed to assess the usefulness of VAS as a tool capturing treatment effect. In addition, it is unclear whether and how VAS could be used to discriminate different levels of asthma control corresponding to the EPR3 guidelines. Such information might have provided further compelling evidence supporting the clinical utility of VAS. Lastly, this study relied primarily on a symptom diary against which changes in VAS scores were compared. Given adolescents’ poor perception of asthma symptoms [27, 37, 38], the sole reliance on the symptom diary may raise a question of validity. To date existing daily symptom monitoring is predominantly self-report in nature. Therefore, comparing one measure of self-report (symptom diary) to another (VAS) is justified to make a case for comparability between two monitoring methods. Nonetheless, further research, such as comparison with daily peak flow, is needed to augment the adequacy of VAS as a daily monitoring tool. Use of VAS in conjunction with peak flow monitoring has been reported to increase symptom perception and medication adherence [39]. Moreover, research establishing the criterion validity of VAS by examining its correlations with airway inflammation (e.g., nitric oxide) and disease burden (e.g., asthma exacerbation, acute healthcare utilization or school absenteeism) is warranted.

Despite the identified limitations, this study has important clinical implications. Demonstrated correlations between the VAS and a daily symptom assessment and an asthma control measure suggest that the single-item VAS be a viable alternative to multiple-question, multiple-choice methods. Inability to identify day to day variations in symptoms often presents challenges for appropriate clinical decision-making. Current retrospective recall methods or multi-item symptom diaries have been found inadequate, due to recall bias or poor patient compliance. In contrast, VAS is a simple, one-step solution to current practice of symptom monitoring, and has the potential to aid tracking daily symptoms in real time without substantially increasing burdens for patients.

## Conclusions

Success in asthma management hinges on patients’ competency to detect and respond to ever-changing symptom severity. Thus, it is crucial to have valid and simple methods of symptom assessment that can be readily incorporated into daily life with minimal patient or provider burden. This study supports VAS as the simplest possible symptom tracking tool for adolescents in ambulatory settings. Future research is warranted to determine the effectiveness and long-term sustainability of VAS and to explore the extent to which daily VAS would facilitate patients’ self-management and providers’ clinical decision making (e.g. medication management). To enhance daily accessibility and adherence, VAS digital versions downloaded onto smart phones can be considered. This will not only facilitate real time, long-term symptom tracking and increase its appeal to adolescent patients but also holds potential for integration with electronic medical records, enhancing clinical usefulness of VAS data.

## Abbreviations

ACD: Asthma Control Diary
EPR3: Expert Panel Report 3
FEV1: forced expiratory volume in one second
SABA: short acting beta agonist
VAS: visual analogue scale
VAS-am: morning VAS
VAS-pm: evening VAS
